# The genome of the Arctic snow alga *Limnomonas spitsbergensis* (Chlamydomonadales)

**DOI:** 10.1093/g3journal/jkae086

**Published:** 2024-04-25

**Authors:** Chris J Hulatt, Hirono Suzuki, Alexandre Détain, René H Wijffels, Thomas Leya, Matthew C Posewitz

**Affiliations:** Faculty of Biosciences and Aquaculture, Nord University, Mørkvedbukta Research Station, 8020 Bodø, Norway; Department of Chemistry, Colorado School of Mines, Golden, CO, 80401, USA; Faculty of Biosciences and Aquaculture, Nord University, Mørkvedbukta Research Station, 8020 Bodø, Norway; Faculty of Biosciences and Aquaculture, Nord University, Mørkvedbukta Research Station, 8020 Bodø, Norway; Faculty of Biosciences and Aquaculture, Nord University, Mørkvedbukta Research Station, 8020 Bodø, Norway; Bioprocess Engineering, AlgaePARC, Wageningen University, Wageningen, 6700 AA, The Netherlands; Fraunhofer Institute for Cell Therapy and Immunology IZI, Branch Bioanalytics and Bioprocesses IZI-BB, Extremophile Research and Biobank CCCryo, 14476 Potsdam-Golm, Germany; Department of Chemistry, Colorado School of Mines, Golden, CO, 80401, USA

**Keywords:** snow algae, climate change, genome assembly, PacBio, IsoSeq, cryophilic, arctic

## Abstract

Snow algae are a diverse group of extremophilic microeukaryotes found on melting polar and alpine snowfields. They play an important role in the microbial ecology of the cryosphere, and their propagation on snow and ice surfaces may in part accelerate climate-induced melting of these systems. High-quality snow algae genomes are needed for studies on their unique physiology, adaptive mechanisms, and genome evolution under multiple forms of stress, including cold temperatures and intense sunlight. Here, we assembled and annotated the genome of *Limnomonas spitsbergensis*, a cryophilic biciliate green alga originally isolated from melting snow on Svalbard, in the Arctic. The *L. spitsbergensis* genome assembly is based primarily on the use of PacBio long reads and secondly Illumina short reads, with an assembly size of 260.248 Mb in 124 contigs. A combination of 3 alternative annotation strategies was used including protein homology, RNA-seq evidence, and PacBio full-length transcript isoforms. The best merged set of annotations identified 18,277 protein-coding genes, which were 95.2% complete based on Benchmarking Universal Single-Copy Orthologs analysis. We also provide the annotated mitogenome, which is a relatively large 77.942 kb circular mapping sequence containing extensive repeats. The *L. spitsbergensis* genome will provide a new resource for research on snow algae adaptation, behavior, and natural selection in unique, low-temperature terrestrial environments that are under threat from climate change.

## Teaser

Snow algae are an important group of photosynthetic microbes living on melting snow and glaciers around the world, but genomic resources are scarce. Here Hulatt et al. assembled and annotated a high quality genome of a Chlamydomonadalean snow alga from the Arctic. The assembly and annotations provide a first glimpse into the evolution and adaptive strategies of terrestrial microeukaryotes that have adapted to life at extremely low temperatures.

## Introduction

Algae inhabiting the surfaces of snow and ice play significant roles in the ecology and biogeochemistry of polar and alpine environments (Leya 2013; Hoham and Remias 2020). These highly pigmented photosynthetic microbes have further large-scale impacts, by increasing the solar energy absorbance of snow and ice surfaces and contributing to accelerated melting (Ganey *et al*. 2017). Snow algae show high biodiversity (Suzuki *et al*. 2023), though by far the most abundant species that form vibrant, colorful surface blooms belong to the order Chlamydomonadales (Volvocales) (Prochazkova *et al*. 2019; Raymond *et al*. 2022).

**Fig. 1.**
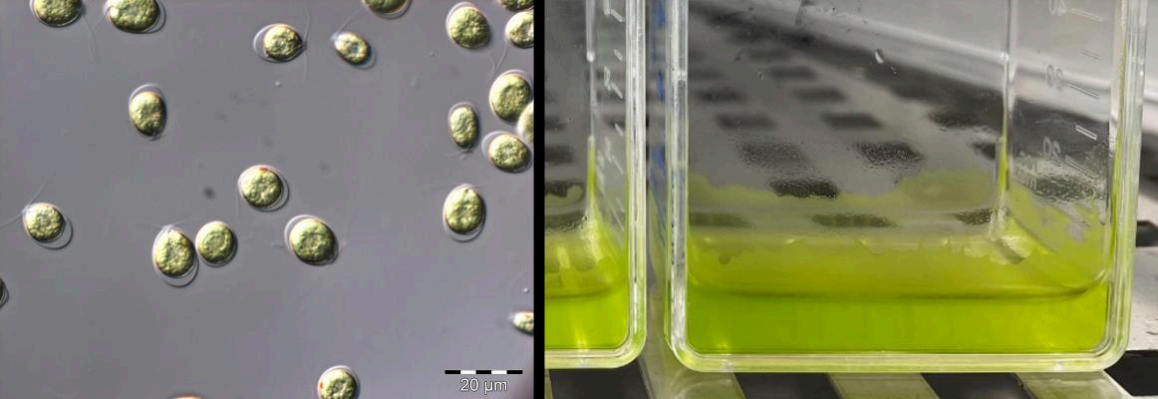
Microphotograph of *L. spitsbergensis* cultures imaged with differential interference contrast microscopy (left). Image of flask cultures incubated at 2°C, showing the migration of motile cells upward through thin surface water films (right).

*Limnomonas spitsbergensis* is a psychrophilic Chlamydomonadalean snow alga that belongs to a newly established genus within the Moewusinia clade, which includes many other Arctic and Antarctic strains, as well as temperate and extremophilic species (Tesson and Pröschold 2022; Tesson *et al*. 2023). Cultures of *L. spitsbergensis* are comprised primarily of biciliate cells that remain highly motile and phototactic at the low temperatures found in snow meltwater, making it an attractive species for studying adaptive snow algae physiology and behavior in the laboratory (Fig. 1). The ability to survive and maintain bioenergetic activity in cryophilic conditions is central to the life history of snow algae and ultimately to their large-scale impacts on masses of snow and ice, yet we lack insight into their adaptation, genome architecture, and evolution.

The isolate of *L. spitsbergensis* sequenced in this study, CCCryo 020-99_CH, was originally collected in 1999 from melting snow on Spitsbergen, Svalbard (79°16′N 11°46′E). High-resolution phylogenetic analysis of the nuclear small subunit ribosomal DNA sequence shows that our sequenced strain is placed with 2 other *L. spitsbergensis* strains in a clade of other *Limnomonas* recently proposed by Tesson and Pröschold (2022) (see Supplementary Fig. a in Supplementary File 1). The objectives of this project were (1) to establish a high-quality genome sequence for a Chlamydomonadalean snow algae, including a comparative overview of the genome architecture with other Volvocine algae, and (2) to provide accurate gene annotations for future research on adaptive mechanisms including protection from freezing, sensing, motility-associated genes, and genome-wide selection effects on snow algae evolution.

## Methods

### Sample preparation

Cultures of *L. spitsbergensis* strain CCCryo 020-99_CH, which is hosted at the Culture Collection of Cryophilic Algae (Fraunhofer Institute IZI-BB, Potsdam, Germany), were prepared by streaking cells on agar medium and selecting colonies. Colonies were transferred and grown in flasks containing 0.2 μm filtered TAP-Medium supplemented with 40 μg mL^−1^ ampicillin. The recipe for TAP-Medium can be downloaded at http://cccryo.fraunhofer.de/sources/files/medien/TAP.pdf. The cultures were verified free of contaminants using flow cytometry and plating on agar medium. For DNA sample preparation, cells were subsequently cultivated in a 500 mL bioreactor sparged with 0.45 μm filtered air at 10°C and an irradiance of 100 μmol photons m^−2^ s^−1^ photosynthetically active radiation. In the late exponential growth phase, cell pellets of ∼1 g fresh weight were collected by centrifugation into 1.5 mL microfuge tubes. Samples for RNA sequencing were initially cultivated in the 500 mL bioreactor and then subsequently split into multiple Erlenmeyer flasks that were exposed to different stress conditions, including cold (2°C), dark, or low-nutrient conditions for 24 h to increase the range of expressed genes. Samples were then pooled together again, collected by centrifuge in 1.5 mL microfuge tubes, frozen in liquid N_2_, and then stored at −80°C.

### Nucleic acid extraction and sequencing

Four sets of nucleic acid samples were extracted and sequenced in this project. PacBio continuous long-read (CLR) DNA extraction and sequencing was performed by Arizona Genomics Institute (AGI, Tucson, Arizona, United States). AGI first harvested nuclei in isolation buffer (10 mM Tris-HCL, pH 8.0, 10 mM EDTA pH 8.0, 100 mM KCL, 0.5 M sucrose, 4 mM spermidine, 1 mM spermine, 20% Triton X-100); then, high molecular weight DNA was extracted using the protocol of Doyle and Doyle (1987) with minor modifications. Nuclei were gently lysed in 2% CTAB buffer containing proteinase K, PVP-40, and beta-mercaptoethanol for 1 h at 50°C. After centrifugation, the supernatant was gently extracted twice with 24:1 chloroform:isoamyl alcohol. Sodium acetate solution (3 M) was added at 10% volume to the upper phase, gently mixed, and DNA precipitated with iso-propanol. DNA precipitate was collected by centrifugation, washed with 70% ethanol, air dried for 5–10 min, and dissolved thoroughly in elution buffer at room temperature followed by RNAse treatment. DNA was further cleaned with magnetic beads into elution buffer. The size of HMW DNA was initially validated with a CHEF-DR II system (BioRad, United States) and then analyzed with pulsed field gel electrophoresis ahead of sequencing library preparation. Twenty micrograms of HMW DNA were sheared to 25–100 kb for the construction of a CLR sequencing library using SMRTbell Express Template Prep kit 2.0. The library was size selected on a Blue Pippin instrument overnight using U1 marker and 30 kb or higher size selection. The recovered final library was quantified with Qubit HS and size checked on a Fragment Analyzer (Agilent, United States). The sequencing library was prepared with PacBio Sequel Sequencing kit v.2.1, loaded to 1 M SMRT cells, and sequenced in CLR mode in the Sequel instrument for 10 h. In total, 4 1 M SMRT cells were sequenced yielding 2.17 million subreads and 38.58 Gigabases (Gb). DNA for Illumina sequencing was extracted with a QIAmp DNA kit (Qiagen LLC, United States), and libraries were prepared using a NEBNext kit from New England Biolabs Inc. (Ipswich, MA, United States). Illumina DNA sequencing was performed by Genewiz LLC (United States) on an Illumina HiSeq 2500 producing 60.34 GB of 250 bp paired end (PE) reads. RNA samples for Illumina sequencing were extracted using Trizol–chloroform and prepared using a Clean and Concentrate miniprep kit (Zymo Research, Irvine CA, United States). Library preparation and sequencing was conducted by NovoGene, United States, with a NEBNext library preparation kit (New England Biolabs Inc. Ipswich, MA, United States) and an Illumina HiSeq 4000 yielding 6.22 Gb of 150 bp PE reads. Illumina DNA and RNA reads were subject to subsequent trimming with TrimGalore, a wrapper for Cutadapt (Martin 2011), followed by quality control with FastQC (Andrews 2010). RNA samples for PacBio isoform sequencing (IsoSeq) were extracted using Trizol–chloroform and precipitated in ethanol to yield high-integrity RNA for full-length transcript sequencing. IsoSeq cDNA library preparation and sequencing was performed by AGI with 2 1M v3 LR SMRT cells and 20 h movies.

### Nuclear genome assembly and polishing

The PacBio CLRs were first prepared with bam2bam, a component of SMRT Link software from PacBio (https://www.pacb.com/support/software-downloads/), to remove adapters and internal control sequences. Due to the gentle DNA extraction procedure, the PacBio CLR data nevertheless included abundant organelle sequences that considerably extended assembly runtime. The organelle sequences were selectively removed by assembling a small subset of the data, identifying the draft organelle contigs, mapping reads to the organelle contigs with blasr (Chaisson and Tesler 2012), and collecting the unmapped nuclear genome reads (see Supplementary Fig. b in Supplementary File 1). After this process, 19.92 Gb of subreads were used for assembly of the nuclear genome. The nuclear genome was assembled with Canu v.1.7.1 (Koren *et al*. 2017) and the settings “minOverlapLength=2000,” “minReadLength=2001,” and “corOutCoverage=100” chosen based on contiguity and Benchmarking Universal Single-Copy Orthologs (BUSCO) completeness. The nuclear genome assembly was subsequently polished with both PacBio and Illumina reads. First, the PacBio subreads were mapped to the assembly using pbmm2 (https://github.com/PacificBiosciences/pbmm2), and the sequences were polished with the ARROW hidden Markov model, a component of SMRT Link software from PacBio. The long-read polishing step was repeated 3 times. Illumina 250 bp PE reads were subsequently mapped to the ARROW-polished contigs with BWA-MEM (Li 2013), and the contigs were polished using Pilon (Walker *et al*. 2014), repeating 3 times. Finally, BWA-MEM was used to map the Illumina 250 bp PE reads to the Pilon-polished contigs; then, the FreeBayes variant caller (Garrison and Marth 2012) was used to perform final polishing, repeated in total 5 times. The key command line code and options used throughout the genome assembly and subsequent annotation process are documented in Supplementary File 2.

### Nuclear genome curation

The “PurgeHaplotigs” pipeline (Roach *et al*. 2018) was used to automatically remove short, duplicated, or spurious contigs from the assembly. PacBio CLRs were first remapped to the assembly using minimap2 (Li 2018), with an average 63× achieved genome coverage. Weakly supported contigs were then identified and removed, producing a revised assembly of 149 contigs and 264.508 Mb. The automatically curated assembly was then reviewed manually using contig size, GC content, and results of a BLAST nucleotide search (Altschul *et al*. 1990) against the National Center for Biotechnology Information (NCBI) nt database. A further 25 small contigs were removed yielding a final curated nuclear genome assembly of 124 contigs and 260.248 Mb which has no detectible contamination from other organisms. Assembly quality was verified at each step with BUSCO v5.1.3 (Seppey *et al*. 2019). A second assembly-independent estimate of the genome size was calculated from the 19-mer profile using Jellyfish v.2.2.6 (Marçais and Kingsford 2011).

### PacBio IsoSeq transcripts

IsoSeq data were prepared with the IsoSeq v3 pipeline (https://github.com/PacificBiosciences/IsoSeq) including circular consensus sequence (CCS) read generation from the raw subreads and selection of full-length nonconcatemer reads, followed by clustering and polishing to yield a set of high-quality transcript isoforms. Polished high-quality transcript isoforms included 43,157 sequences of L50 length 1.595 kb.

### Repeat annotation

A de novo library of genomic repeat families was constructed with RepeatModeler2 (Flynn *et al*. 2020), which was run with the extended LTRharvest pipeline for discovery of long terminal repeat retrotransposons (LTR-RTs) (Ellinghaus *et al*. 2008). RepeatMasker was used to determine the repeat elements throughout the genome (Smit et al. 2015). In addition, Tandem Repeat Finder (Benson 1999) was run independently using the settings “2 7 7 80 10 50 500 -d -m -h” to identify simple repeats, and a merged nonredundant repeat annotation was used to soft-mask the genome before coding sequence (CDS) annotation.

### CDS structural annotation

Genome CDS structural annotation was conducted using 3 different methods followed by selection of the best transcript models: (i) gene prediction was conducted using the BRAKER1 pipeline with 150 bp PE RNA-seq evidence (Hoff *et al*. 2016). RNA-seq reads were mapped to the genome using the splice aware aligner STAR (Dobin *et al*. 2013), and braker.pl was run with the “--softmasking” option, allowing alternative transcripts for each gene. (2) Gene prediction was conducted with the BRAKER2 pipeline using alignment of proteins primarily from distantly related organisms using ProtHint (Brůna *et al*. 2021). The protein database comprised the OrthoDB v10.1 viridiplantae orthologs with the proteins from the *Chlamydomonas reinhardtii* v5.5 genome assembly. (3) The third annotation leveraged the IsoSeq data, where the polished high-quality transcripts were aligned to the genome with minimap2 and the option “ax splice:hq” for the spliced alignment of PacBio CCS reads (Li 2018). Aligned transcripts were collapsed into unique isoforms using “collapse_isoforms_by_sam.py” from cDNA Cupcake (https://github.com/Magdoll/cDNA_Cupcake). Gene models were subsequently determined using GeneMarkS-T (Tang *et al*. 2015). The 3 sets of annotations were compared and the best transcript models were selected with TSEBRA (Gabriel *et al*. 2021) using the default configuration settings in the “long_reads” branch for IsoSeq data (https://github.com/Gaius-Augustus/TSEBRA).

### CDS functional annotation

Protein-coding genes were functionally annotated using DIAMOND searches (Buchfink *et al*. 2021), InterProScan-5 (Jones *et al*. 2014) and eggNOG pathway mapping for orthology assignment (Huerta-Cepas *et al*. 2017). The DIAMOND search used the RefSeq Viridiplantae database to assign protein names, to which additional ontology terms from InterProScan and eggNOG results were mapped and combined into a single set of annotations with OmicsBox v3.1.11.

### Mitogenome assembly and annotation

The organelle genomes were assembled independently of the nuclear genome with random subsets of CLRs using Canu v.2.0. The organelle contigs could be clearly identified their size and GC content and were confirmed circular by overlapping sequences at each end. The mitogenome and plastid genomes both assembled into single contigs, but despite testing alternative assemblies and polishing steps, the result for the plastid (>400 kb) did not converge on a unique finished sequence. Thus, we annotated and present the mitogenome assembly in the current work. The mitogenome sequence was subsequently finished with the PacBio command line tools by realigning CLRs with pbmm2 and the option “--preset SUBREAD,” then polishing with VariantCaller and “--algorithm arrow.” Remapping and polishing steps were conducted twice, finally using 500-fold polishing depth to deliver very high consensus accuracy. The finished mitogenome sequence was checked by aligning Illumina 250 bp PE reads, but no further changes were made.

Mitochondrial protein-coding and ribosomal genes were identified with MFannot (Beck and Lang 2010) then refined using BLAST, full-length transcripts, or short RNA sequence reads together with RNAweasel (Lang *et al*. 2007) for placement of introns. Aragorn was used to annotate tRNAs (Laslett and Canback 2004). Repetitive sequences were annotated with tandem repeats finder and ROUSFinder.py (https://github.com/flydoc2000/ROUSfinder). The mitogenome sequence can be found in NCBI GenBank under accession number OQ504168.

Phylogenetic analysis of the *L. spitsbergensis* mitogenome was performed with sequences from 32 related Chlamydomonadalean algae using 7 protein-coding genes (*cox1*, *cob*, *nad1*, *nad2*, *nad4*, *nad5*, and *nad6*) common to all strains. Sequences from 2 additional non-Chlamydomonadalean species were included as the outgroup. Amino acid sequences were aligned with MUSCLE (Edgar 2004), concatenated, and then curated with GBlocks (Talavera and Castresana 2007). The optimal tree based on BIC was constructed with the LG + G + I + F maximum likelihood (ML) model using 1,000 bootstrap replicates in MEGA X v.10.1.8. Secondary branch support values were calculated with MrBayes 3.2.6 (Huelsenbeck and Ronquist 2001) using the Poisson model and gamma rate variation. In total 1,100,000 Markov chain Monte Carlo generations were run over 4 chains; the first 100,000 chains were removed as burnin.

### Comparative genome analysis and statistics

The genome architecture of *L. spitsbergensis* was compared with 13 genomes of related Volvocine algae obtained from NCBI GenBank. To compare the repeat content of the genomes, RepeatModeler2 and RepeatMasker were run on each of the assemblies. The genome assembly CDS were analyzed with OrthoFinder2 (Emms and Kelly 2019) to identify single-copy orthologs and construct a whole-genome phylogeny. The genome sequences were *Astrephomene gubernaculifera* (GCA_021605115.1), *C. reinhardtii* (GCA_000002595.3), *C. incerta* (GCA_016834605.1), *C. schloesseri* (GCA_016834595.1), *C. priscuii* UWO241 (GCA_016618255.1), *Chlamydomonas* sp. ICE-L (GCA_013435795.1), *C. eustigma* (GCA_002335675.1), *Dunaliella salina* (GCA_002284615.2), *E. debaryana* (GCA_016858145.1), *Haematococcus lacustris* (GCA_003970955.1), *Volvox africanus* (GCA_019650175.1), *Volvox carteri* (GCA_000143455.1), and *Volvox reticuliferis* (GCA_019650235.1).

The relationship between genomic repeat elements and genome assembly size was modeled with phylogenetic linear regression using the R package “phylolm” (Ho and Ané 2014) using the species tree from OrthoFinder2.

## Results and discussion

### Nuclear genome assembly

The *L. spitsbergensis* nuclear genome comprises a high-contiguity 260.248 Mb assembly in 124 contigs (Table 1), reflecting the use of long PacBio reads with an L50 sequence read length of 32.946 kb and maximum sequence read length 127.141 kb for assembly (see Supplementary Fig. b in Supplementary File 1). The quality of the genome was monitored with BUSCO in “--genome” mode from the raw Canu assembly through polishing with CLR and Illumina reads, to final curation. The polishing process with both PacBio and Illumina reads had a negligible effect on the assembly size or the BUSCO results, and the subsequent curation and quality control process improved the assembly by removing small redundant sequence fragments (see Supplementary Table a in Supplementary File 1). The genome size of the finished *L. spitsbergensis* assembly is supported by independent analysis of the 19-mer profile, which estimates a genome of 232.957 Mb (see Supplementary Fig. c in Supplementary File 1). With an average GC content of 54.15%, the *L. spitsbergensis* nuclear genome sits centrally within the expected range of Chlamydomonadalean algae, which varies from the 44.8% GC genome assembly of *C. eustigma* (GCA_002335675.1) to the 67.1% GC assembly of *E. debaryana* (GCA_016858145.1).

**Table 1.** *L. spitsbergensis* genome assembly statistics.

Feature	Statistic
Genome assembly size	260,248,090 bp
Number of contigs	124
Contig L50	3.933 Mb
Contig N50	21
Max contig length	10.736 Mb
GC content (±contig)	54.15 ± 0.68%

### Repetitive DNA sequences

Analysis with RepeatModeler2 and RepeatMasker identified 33.16% of the *L. spitsbergensis* genome as repetitive. The results shown in Table 2 indicate the prevalence of retroelements among the various classes of interspersed repeats. Nine percent of the *L. spitsbergensis* genome was comprised of retroelements, of which LTR-RTs retrotransposons comprised 5.96% of the sequences. The majority of LTR elements in the genome was of the Gypsy/DIRS1 type (4.41%), with fewer BEL/Pao family elements (0.91%) and no annotated Copia LTR elements.

**Table 2.** Interspersed repeats in the nuclear genome of *L. spitsbergensis*. Repeat elements that were not found in the assembly have been omitted.

Name	Number	Length (bp)	Percent (%)
**Retroelements**	43,503	23,425,427 bp	9.00
SINEs	149	17,188 bp	0.01
LINEs	21,057	7,894,454 bp	3.03
RTE/Bov-B	12,066	3,801,489 bp	1.46
L1/CIN4	755	338,258 bp	0.13
LTR elements	22,297	15,513,785 bp	5.96
BEL/Pao	7,196	2,369,491 bp	0.91
Gypsy/DIRS1	8,414	11,483,832 bp	4.41
**DNA transposons**	3,715	2,544,966 bp	0.98
Hobo-Activator	2,114	1,146,375 bp	0.44
Tc1-IS630-Pogo	908	1,261,481 bp	0.48
**Rolling circles**	989	509,834 bp	0.20
**Unclassified**	242,500	59,288,265 bp	22.78
**Total interspersed repeats**		85,258,658 bp	32.76
**Small RNA**	668	288,809 bp	0.11
**Satellites**	888	250,671 bp	0.10
**Simple repeats**	72	2573 bp	<0.01

### Comparative gene annotation using RNA-seq, proteins, and IsoSeq data

Annotation of the nuclear genome using RNA-seq evidence (BRAKER1) identified 22,993 transcript models derived from 21,410 genes, while protein-based training (BRAKER2) annotated a comparable 21,371 transcripts from 20,252 genes (Table 3). Annotation using the IsoSeq full-length transcripts yielded 29,987 transcript models from 11,428 genes, representing greater transcript diversity from fewer gene loci. BUSCO analysis of CDS in mode “--transcript” showed that the BRAKER1 and secondly BRAKER2 workflows identified the most complete set of CDS, while the IsoSeq annotation offered improved accuracy, evidenced by reduced fragmentation, at the cost of incomplete annotation across the genome. IsoSeq annotations yielded CDS sequences with on average slightly fewer exons and slightly shorter introns than the BRAKER pipelines, indicating the value of direct, full-length transcript evidence for describing gene structures and splice sites. The final CDS structural annotations that were merged and selected with TSEBRA comprised 25,023 transcripts derived from 18,277 gene loci, including 95.2% complete, 0.5% fragmented, and 4.3% missing genes based on BUSCO analysis with the “chlorophyta_odb10” set of 1,509 reference sequences.

**Table 3.** *L. spitsbergensis* genome CDS structural annotation summary using the merged best transcript models selected from independent annotations using BRAKER1, BRAKER2, and IsoSeq annotation pipelines.

Feature	BRAKER1	BRAKER2	IsoSeq	TSEBRA (merged)
Genes	21,410	20,252	11,428	18,277
Transcripts	22,993	21,371	29,987	25,023
Transcript L50	2,478 bp	2,475 bp	1,674 bp	2,152 bp
Mean CDS length	1,676 bp	1,740 bp	1,367 bp	1,406 bp
Mean exons per CDS	7.9	7.4	6.0	6.7
Mean intron length	823 bp	796 bp	720 bp	788 bp
BUSCO complete	96.2%	89.1%	75.9%	95.2%
BUSCO duplicated*^a^*	20.3%*^a^*	19.6%*^a^*	41.3%*^a^*	37.2%*^a^*
BUSCO fragmented	1.1%	1.7%	0.5%	0.5%
BUSCO missing	2.7%	9.2%	23.6%	4.3%

BUSCO was run using all transcript isoforms with the chlorophyta_odb10 reference set in transcript mode.

^*a*^Note that duplicated BUSCOs in this table include alternative transcript isoforms from the same genomic loci.

### Genome phylogeny and architecture

**Fig. 2.**
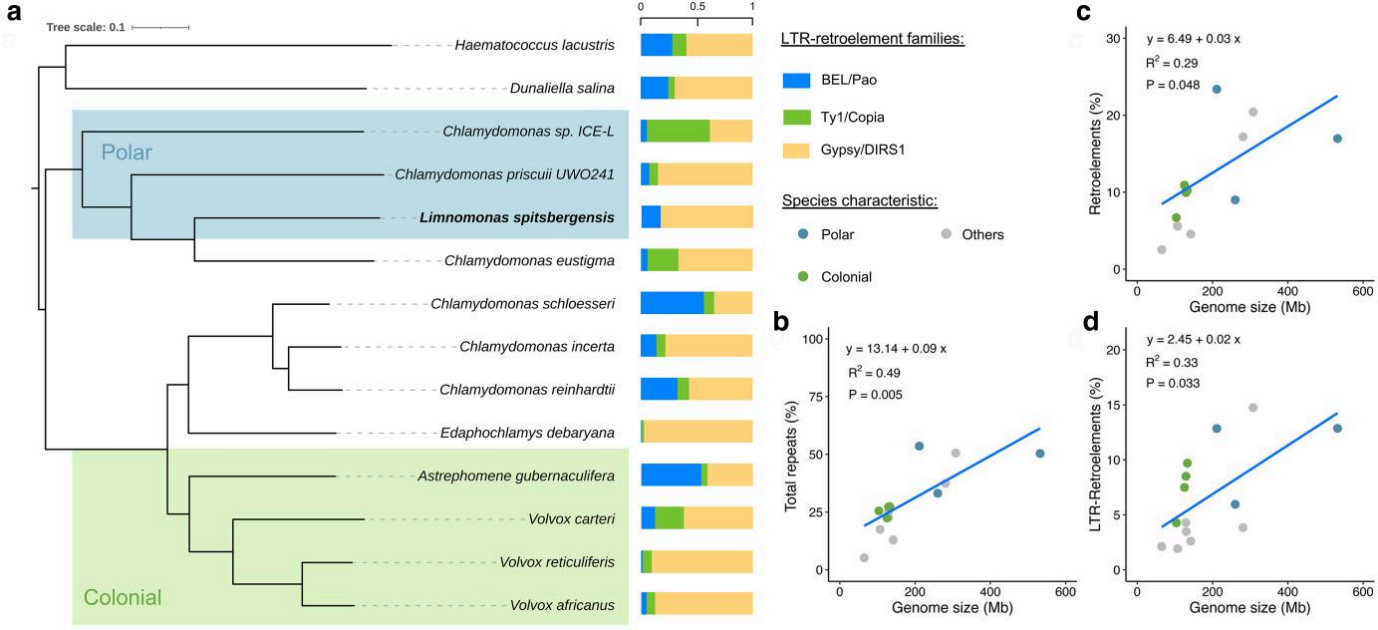
a) Whole-genome phylogeny of 14 sequenced Volvocales, including *L. spitsbergensis*, inferred from all orthogroups with OrthoFinder2. The proportion of each of 3 LTR-retroelement families in each genome assembly is indicated. b) Correlation between genome size (Mb) and total % genomic repeats. c) Correlation between genome size (Mb) and total retroelements (%). d) Correlation between genome size (Mb) and LTR-retroelements (%). All repetitive element data were provided by de novo analysis using RepeatModeler2.

The genome assemblies and CDS of 13 related Volvocine green algae were obtained from NCBI and used to explore the phylogeny and compare the genome architecture of *L. spitsbergensis* with related Chlamydomonadalean algae. Whole-genome phylogeny prepared with OrthoFinder2 placed *L. spitsbergensis* in a clade with 2 Antarctic *Chlamydomonas* strains, ICE-L, and UWO 241, together with *C. eustigma* NIES-2499, which is a temperate species isolated from acid mine drainage in Japan (Fig. 2a). Although both ICE-L and UWO241, like *L. spitsbergensis*, inhabit polar environments, the Antarctic strains were isolated respectively from sea ice and Lake Bonney, a saline and permanently ice-covered lake (Zhang *et al*. 2018; Kalra *et al*. 2020).

At 2.4 times the size of the *C. reinhardtii* assembly, the genome of *L. spitsbergensis* is among the larger Volvocine algal genomes assembled to date, comparable in size with the 281 Mb *D. salina* assembly. Yet, larger Volvocine genomes have been assembled, including the 532 Mb assembly of *Chlamydomonas* ICE-L and the 308 Mb assembly of *H. lacustris*. The expansion of plant genome size is linked to the proliferation of repeats (Schley *et al*. 2022), especially LTR-RTs, so we examined the correlation of interspersed repeat elements with the genome assembly size within the Volvocales (Fig. 2b–d). Total interspersed repeats ranged from 5 to 54% of the genome sequence and correlated (*R*^2^ = 0.49, *P* = 0.005) with the assembly size (Mb). Retroelements and LTR-retroelements in the assembly (%) each also correlated significantly with the assembly length (Mb), describing overall patterns of increasing transposable element abundance with increasing genome size.

Zhang *et al*. (2020) described the LTR-RTs in the *Chlamydomonas* ICE-L genome, identifying *Copia* and *Gypsy* elements as the primary superfamilies. Despite the overall correlation between repeat elements and genome size expansion, our analysis applying RepeatModeler2 shows substantial variation in 3 LTR-RT superfamilies across the Volvocales, with little evidence of association between phylogeny and the families of LTR-retroelements that are present.

### Mitochondrial genome

**Fig. 3.**
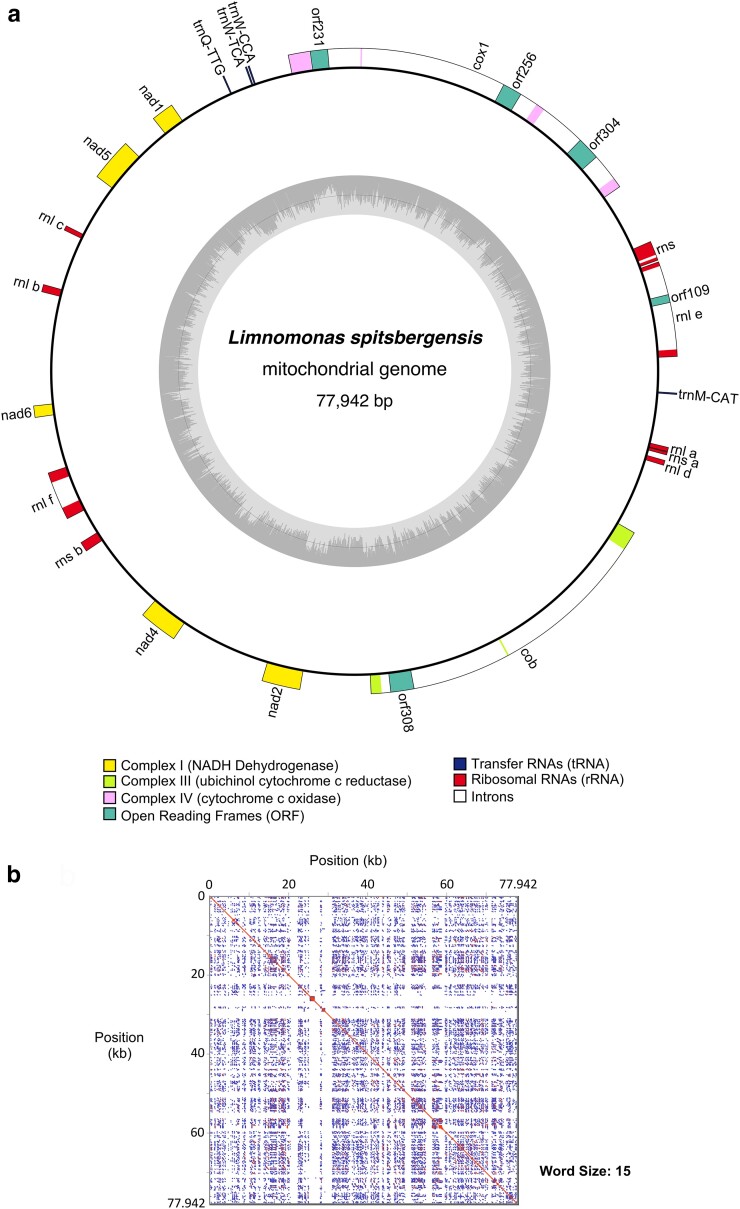
a) Mitochondrial genome map displaying the position and structure of 7 protein-coding genes, ribosomal RNA genes, and tRNAs in the circular mapping sequence. Group I introns are shown, with the position of their respective homing endonucleases indicated by open reading frames. b) The dotplot visualizes repetitive sequences across the whole 77,942 bp mitogenome.

The mitochondrial genome of *L. spitsbergensis* is a 77,942 bp circular mapping sequence with a 48.8% GC content (Fig. 3a). It is substantially larger than most sequenced Chlamydomonadalean mitogenomes (∼13 to 46 kb), yet smaller than that of *H. lacustris* NC_044670 (124.6 kb) and a novel nonphotosynthetic *Polytoma*-like flagellate OM479424 (104.8 kb) (Del Vasto *et al*. 2015; Zhang *et al*. 2019; Pánek *et al*. 2022). The rRNA genes of *L. spitsbergensis* are scattered around the mitogenome, akin with other Chlamydomonadalean algae where the mitoribosome is constructed from multiple fragmented rRNAs (Waltz *et al*. 2021).

**Fig. 4.**
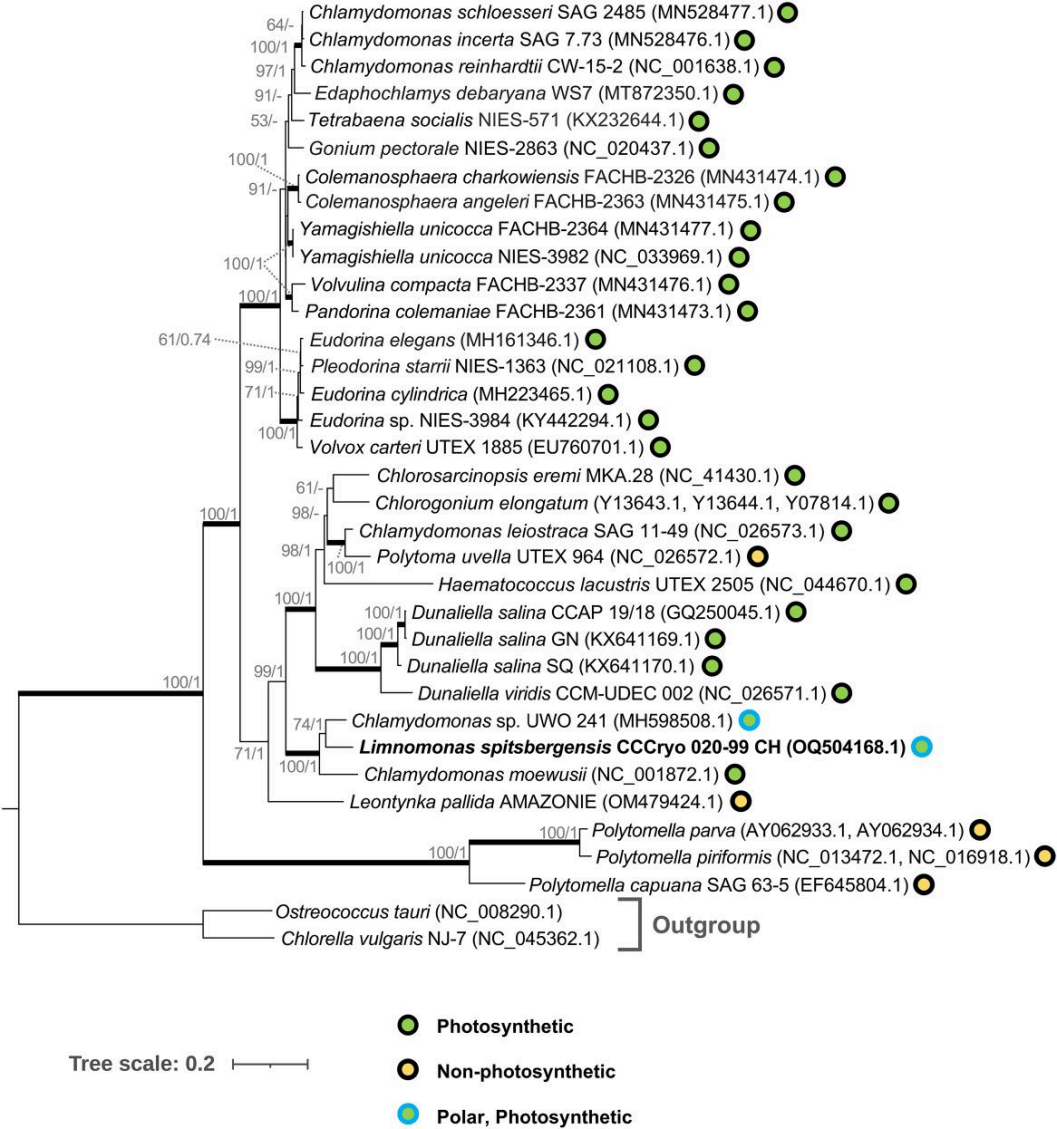
Phylogenetic analysis of the *L. spitsbergensis* mitogenome (bold) with 32 related Chlamydomonadalean algae using 7 protein-coding genes (*cox1*, *cob*, *nad1*, *nad2*, *nad4*, *nad5*, and *nad6*) common to each strain. Two non-Chlamydomonadalean chlorophytes, *Ostreococcus tauri* and *Chlorella vulgaris*, were included as the outgroup. The optimal tree based on BIC was constructed with the LG + G + I + F ML model using 1,000 bootstrap replicates. Secondary branch support values were calculated with MrBayes 3.2.6 (Huelsenbeck and Ronquist 2001) and are displayed in format “ML bootstrap/MrBayes.”

Seven protein CDS found in the *L. spitsbergensis* mitogenome are typical of Chlamydomonadalean algae comprising *cob*, *cox1*, *nad1*, *nad2*, *nad4*, *nad5*, and *nad6*. Phylogenetic analysis places *L. spitsbergensis* adjacent to *Chlamydomonas* sp. UWO 241, an Antarctic strain, and secondly to *Chlamydomonas moewusii*, a temperate species, within a clade with full statistical support (ML/BL:100/1.00) (Fig. 4). Although both polar species are placed together, cryophilic traits have evolved independently across diverse protist lineages (Dorrell *et al*. 2023), including among Chlamydomonadalean algae (Cvetkovska *et al*. 2017), and further sampling of Arctic and Antarctic species mitogenomes is needed to resolve fine-scaled patterns in species ecophysiology and biogeography.

Group I introns in the *cob*, *cox1*, and *rnl-e* genes harbor in total 5 homing endonuclease family genes (HEGs). Intron-encoded HEGs are indicated by open reading frames, but their start codons are typically difficult to place, and weak alignment of their protein sequences indicates disparate origins. Introns, including their endonucleases, account for 17,578 bp (22.6%) of the mitogenome sequence and thus contribute substantially to overall mtDNA size.

Repeat sequences are prolific in intergenic regions (Fig. 3b and Supplementary Fig. d in Supplementary File 1). Tandem repeats comprise 5,981 bp (7.7%) of the sequence, while nontandem repeats comprise 29,826 bp (38.3%). A merged and nonredundant repeat annotation accounts for 31,557 bp (40.5%) of the sequence, with a GC content of 55.8%.

The mitogenome contains 4 tRNAs, 3 of which encode trnM(cat), trnW(cca), and trnQ(ttg) that are common to many other Chlamydomonadalean mitogenomes. The 4th tRNA recognizes the UGA codon, which is found within multiple CDS and is supported by IsoSeq full-length transcript evidence (see Supplementary Sequence a in Supplementary File 1). To determine the amino acid encoded by UGA, protein sequences of the 32 related Chlamydomonadalean algae shown in Fig. 4 were aligned with gene and transcript sequences from *L. spitsbergensis*, and their frequency was determined across 5 genes and 11 positions (see Supplementary Fig. e and Table b in Supplementary File 1). The majority (88.4%) of internal UGA codons in *L. spitsbergensis* corresponds to Trp, so consistent with this evidence, the 4th tRNA was annotated trnW(tca). Mitochondrial UGA stop-to-sense (stop→Trp) codon reassignment has been previously identified in Prasinophytes (Noutahi *et al*. 2019) and more widely across eukaryotes, but this appears to be the first evidence in the Chlamydomonadales.

## Conclusion

Here, we present the first high-quality genome of a cryophilic Chlamydomonadalean snow alga from the Arctic, annotated with multiple sets of gene evidence including full-length IsoSeq transcripts. The results will enable further studies on the unique physiological adaptations of these extremophilic microbes and allow us to study the regulation of key genes involved with snow algae survival and behavior at extremely low temperatures.

The *L. spitsbergensis* nuclear genome is among the larger Volvocale genomes sequenced to date and falls along the overall pattern of repeat and retroelement-driven genome size expansion in the class. The *L. spitsbergensis* genome contiguity and accuracy benefits from our use of long PacBio CLR reads at sufficient sequencing depth, which effectively span repeats and provide subsequent high base polishing accuracy. Following the pattern observed in other Volvocales (Smith *et al*. 2013; Featherston *et al*. 2016; Zhang *et al*. 2019), the mitogenome of *L. spitsbergensis* is also enlarged by repeat sequences.

Compared with their marine counterparts, terrestrial snow and ice algae have unique ecology. Snow and glacier systems are characterized by bright sunlit surfaces comprised of thin, fresh, meltwater films where cells must survive not only low temperatures but dynamic freeze–thaw cycles and very low-nutrient levels (Hoham and Remias 2020). The *L. spitsbergensis* genome will provide insight into natural selection and genome evolution under multiple forms of physiological stress and assist in explaining the propagation and dispersal of algae across the surfaces of snow and ice. As the world warms, such data will be invaluable for understanding the responses of polar and alpine microbes and predicting feedbacks with climate change.

## Supplementary Material

jkae086_Supplementary_Data

## Data Availability

The genome sequence data that support the findings of this study are openly available in NCBI GenBank at https://www.ncbi.nlm.nih.gov/genbank/. The genome sequence has GenBank accession number JAYJMM000000000. The associated **BioProject**, **SRA**, and **Bio-Sample** numbers are PRJNA953517, SRR24109343 and SRR24109344, and SAMN34118326, respectively. The mitogenome is available separately under OQ504168. The genome sequence, GFF annotation, and functional annotation files are hosted at Zenodo under doi 10.5281/zenodo.10478697. Supplemental  material available at G3 online.
