# Supplementary material for: The genome of the Arctic snow alga Limnomonas spitsbergensis (Chlamydomonadales)
Source: G3 (Bethesda). 2024 Apr 25;14(7):jkae086. doi: 10.1093/g3journal/jkae086 (PMC11228838; doi:10.1093/g3journal/jkae086)
Supplement: jkae086_Supplementary_Data [file jkae086_supplementary_data.zip › Supplementary_File_S1.pdf]

The genome of the Arctic snow-alga *Limnomonas spitsbergensis* (Chlamydomonadales)

Chris J. Hulatt[1,2]*, Hirono Suzuki[1], Alexandre Détain[1], René H. Wijffels[1,3], Thomas Leya[4], Matthew C. Posewitz[2]

[1]Faculty of Biosciences and Aquaculture, Nord University, Mørkvedbukta Research Station, 8020 Bodø, Norway.

[2]Department of Chemistry, Colorado School of Mines, Golden, CO, 80401, USA.

[3]Bioprocess Engineering, AlgaePARC, Wageningen University, PO Box 16 Wageningen, 6700, AA, The Netherlands.

[4]Fraunhofer Institute for Cell Therapy and Immunology IZI, Branch Bioanalytics and Bioprocesses IZI-BB, Extremophile Research and Biobank CCCryo, 14476 Potsdam-Golm, Germany.

*Corresponding Author: christopher.j.hulatt@nord.no; (+47) 9009-8564

Snow algae genome

SUPPLEMENTARY FIGURES

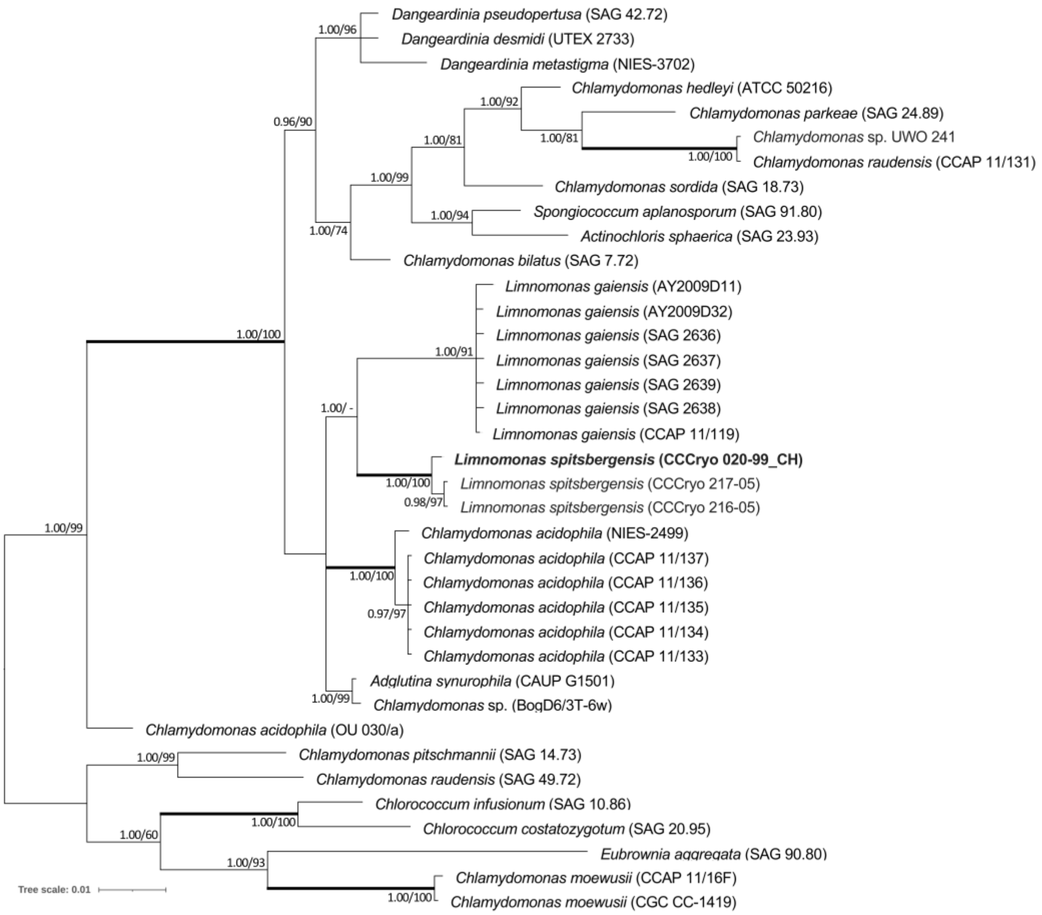

Figure A. Phylogeny of the Moewusii clade (Chlamydomonadales) including the sequenced strain of *Limnomonas spitsbergensis* (CCCryo 020-99_CH, bold) using 18S rDNA sequences. The tree was constructed with MrBayes (Huelsenbeck & Ronquist 2001) using 1,656 bp of aligned positions across 37 taxa. Maximum likelihood (ML) phylogeny was constructed with the K2+I+G model, selected as the best model as calculated by MEGA X 10.1.8. Numbers next to branches indicate statistical support values [Bayesian posterior probabilities/ML bootstraps (1000 replicates)]. Thick lines indicate the branches with full statistical support (BI/ML:1.00/100). Sequences, aside from 020-99_CH, are as found in Tesson & Pröschold (2022).

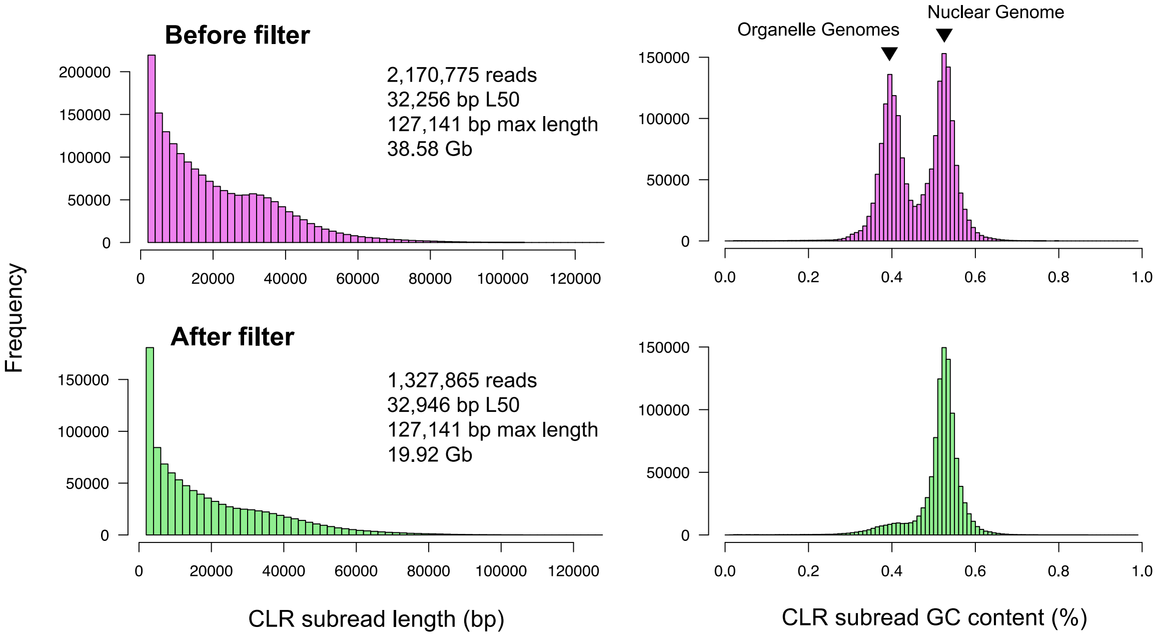

Figure B. Histograms display the length (bp) and GC content (%) of the PacBio CLR subreads that were used for assembly and polishing. The upper panels (pink) show data from all of the subreads from the four sequenced SMRT cells. The lower panels (green) show the length and GC content after using blasr alignment to selectively remove the organelle sequences. The selective removal of the low-GC organelle peak is shown, whilst the abundance, length and N50 of the nuclear genome reads is preserved.

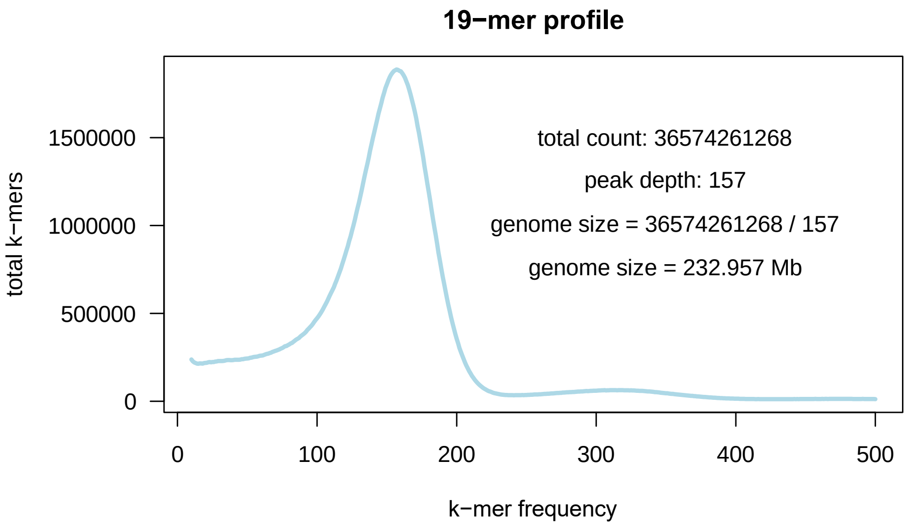

Figure C. Genome size estimation using k-mer analysis of the Illumina 250 bp PE DNA sequence reads. The 19-mer profile was calculated with Jellyfish v.2.2.6 where the total 19-mers numbered 36,574,261,268 and the peak depth was 157-fold. The estimated genome size was thus 232.957 Mb.

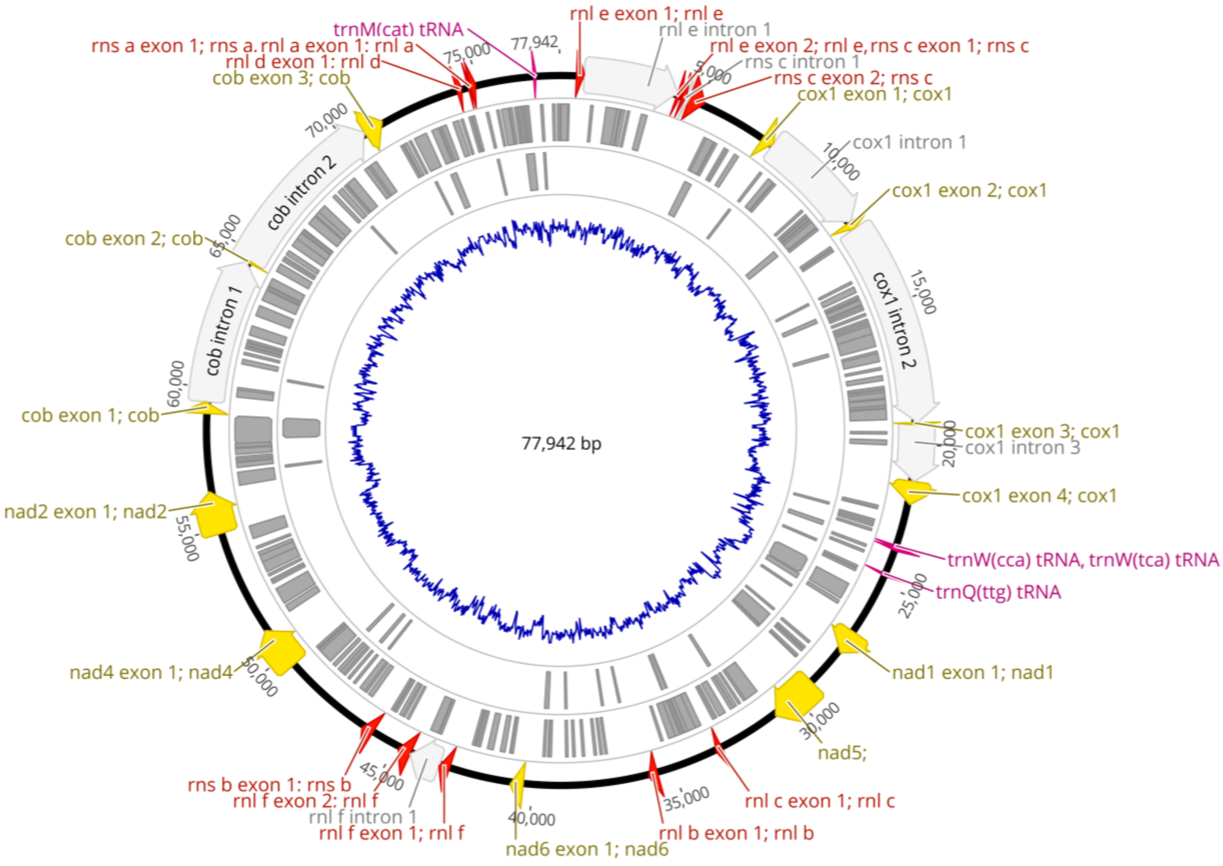

Figure D. Annotated mitogenome displayed with additional repeat tracks (dark grey). The innermost grey track represents tandem repeats identified by Tandem Repeat Finder, where overlapping repeats are merged. The outer grey track displays non-tandem repeat regions annotated by ROUSFinder.py, and overlapping regions are merged.

Position 1

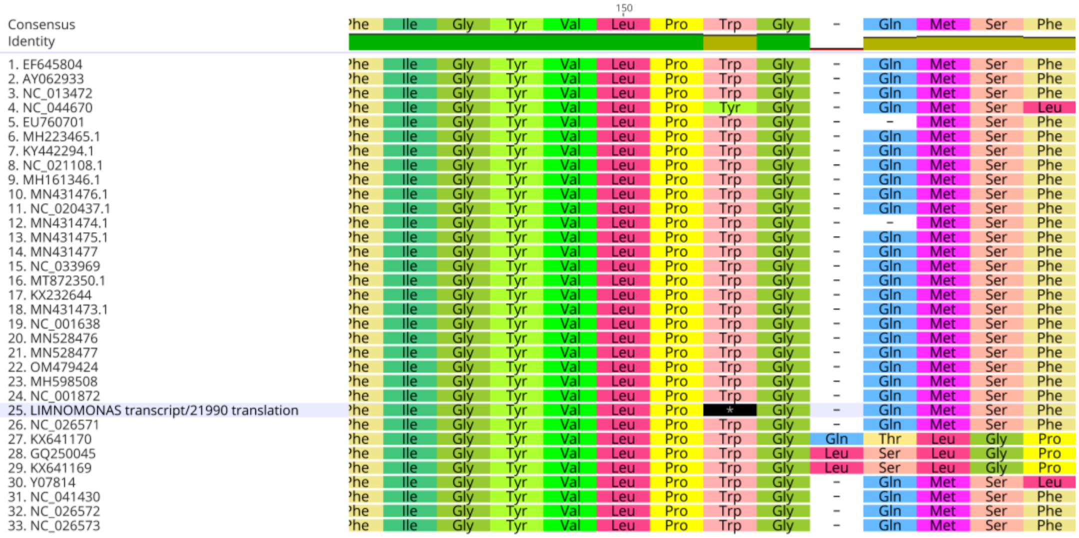

Position 2

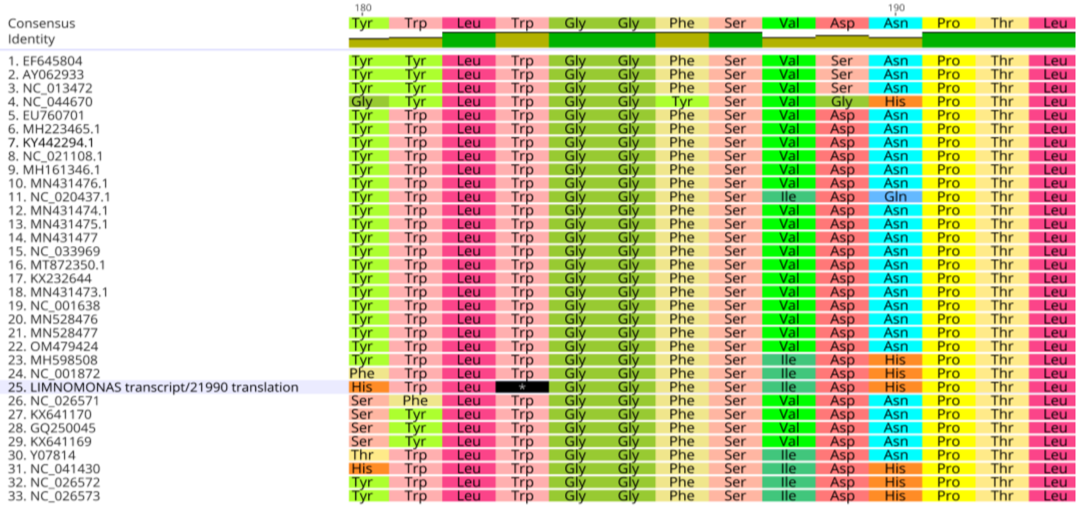

Figure E. Protein sequences of the *cob* gene from the 32 related Chlamydomonadalean algae mitogenomes shown in manuscript Figure 4, aligned with the translated iso-seq transcript "transcript/21990" corresponding to the annotated *cob* gene of *Limnomonas spitsbergensis*. Two UGA positions in the *cob* gene are marked "*" by standard translation table that were subsequently allocated to Trp. The alignments show that 31/32 and 32/32 of the amino acids corresponding to the UGA codons are Trp at each position, respectively. Sequences from the 32 related Chlamydomonadalean mitogenomes are indicated by their accession numbers.

SUPPLEMENTARY TABLES

Table A. BUSCO scores and assembly statistics through the genome preparation workflow from the raw Canu assembly to the fully polished assembly (Arrow + Pilon + FreeBayes) and the final curated genome (PurgeHaplotigs + manual curation). BUSCO v5.1.3 was run with the following options: “--augustus”, “--long”, “--genome” and “-l chlorophyta_odb10” to use the set of 1,519 chlorophycean reference genes.

Assembly Stage	Size (MB)	Contigs (*n*)	N/L50	BUSCO result
Raw Canu Assembly	279.626	361	24/3.756	C:96.5% [S:81.9%, D:14.6%]<br>F:0.5%<br>M:3.0%
Polished Assembly<br>(Arrow + Pilon + FreeBayes)	279.862	361	24/3.757	C:96.6% [S:81.6%, D:15%]<br>F:0.4%<br>M:3.0%
Final curated<br>(PurgeHaplotigs + manual)	260.248	124	21/3.933	C:95.0% [S:81.4%, D:13.6%]<br>F:0.5%<br>M:4.5%

Table B. Counts of amino acids at aligned positions corresponding to the UGA codon in *Limnomonas spitsbergensis* using sequences from the 32 related Chlamydomonadalean mitogenomes shown in manuscript Figure 4. A total of 11 positions are found in five of the protein-coding genes, at which the majority (88.4%) encode Trp. Sequences were aligned with MUSCLE and visualized in Geneious Prime 2022.2.2, as illustrated for the *cob* gene in Supplementary Figure E.

**Gene**	**Position**	**Amino Acid**								
		**Trp**	**Tyr**	**Leu**	**Phe**	**Ile**	**Val**	**Ala**	**"-"**	**Total**
cob	Pos1	31	1	0	0	0	0	0	0	32
	Pos2	32	0	0	0	0	0	0	0	32
cox1	Pos1	28	4	0	0	0	0	0	0	32
	Pos2	32	0	0	0	0	0	0	0	32
	Pos3	32	0	0	0	0	0	0	0	32
	Pos4	28	3	0	0	0	0	0	1	32
	Pos5	23	4	3	0	1	1	0	0	32
nad1	Pos1	29	3	0	0	0	0	0	0	32
nad2	Pos1	20	3	5	1	2	0	1	0	32
	Pos2	29	3	0	0	0	0	0	0	32
nad5	Pos1	27	1	0	4	0	0	0	0	32
	**Total**	311	22	8	5	3	1	1	1	352
	**%**	88.4	6.3	2.3	1.4	0.9	0.3	0.3	0.3	

SUPPLEMENTARY SEQUENCES

Sequence A. Internal UGA codons were found within multiple CDS, and the mitogenome has a fourth tRNA that recognizes the UGA codon. Iso-seq CCS (circular consensus sequence) transcript read data provides unambiguous confirmation of gene sequences. A full-length high quality CCS sequence of the mitochondrial *cob* gene transcript contains two internal UGA codons.

```
>transcript/21990
ATGCGTTTACATAATAGAGTACAAGTTTTGAAGTTATTACAAAACCATTTAGGAGTATATCCAACACCAATGAACCTTA
ATTGGAATTGGAGTTGGGGTTCTTTATCCGGAATCTTATTAGGTAGTCAAATGATTACAGGTATATTACTTGCTTGTC
ACTACGTAGGTCATGTAGATCATGCCTTTGCTAGTGTCCAACATTTAATGGTAGATGTACCATCCGGTTTAATTTTAC
GCTATACCCATGCTAATGGGGCTAGTTTATTTTTCACTGTAGTATATTTACATGTTTTACGTGGAATTTATTATAGCAG
TGGTAACCAACCACGTGAATTAGTATGGATAGTGGGTGTAGTTATTTTATTATTAATGATTATAACAGCCTTCATTGGT
TACGTGCTGCCTTGAGGTCAAATGTCTTTTTGGGGCGCGACCGTAATAACAAGTTTAGCTACAGTAATACCCGTTG
TAGGAAAGGAAATAGTTCATTGGTTATGAGGAGGTTTCAGCATAGATCATCCAACCTTAAACCGTTTTTATAGTTTCC
ATTACACTTTACCATTTATATTAGCTGGTTTAAGTATTTTTCATATCGCAGCATTACACCAATATGGTAGTACTAATCCA
TTAGGTATTAATACTCAAAGCAGCACTATTCATTTTGGTACTTATTTTGCTAGTAAAGATTTATTAGCACTTTTATTTTT
ATTATTAGTGTTTGCTATTTTAGTCTTTTTCTATCCTGAATATTTAGCTCATCCCGATAATTTAATACCAGCTAATCCTTA
TTCTACACCTCAACACATTGTACCCGAATGGTATTTCTTGTGGGTTTATGCTATATTACGTAGTATTCCTAATAAAGCT
GTAGGGGTTATTGCGATTGGTTTAGTATTTGCCAGTTTAATAAGCTTACCTTTTTTAAGTGTTGTACAAGTAGGATCT
CCTCGTTTTCGCGTCATATACGAACGTTTATTCTGGATCTTTGTTGCTGACGTTTTCTTATTAACTTGGTTAGGAGGT
CAAGAAATAGCAGAACCTACTGTTCTTATGGGACAAATATGCACTGTAATCTTTTTTGTTTATCTCTTAGTATTATTAC
CTTTCTTAGGTTGGATTGAAACCGCTTTAATTTTAGCTTAATCTTATCCATTTTAATATACAACCCCCCCCGC
```
