# Supplementary material for: The genome of the Arctic snow alga Limnomonas spitsbergensis (Chlamydomonadales)
Source: G3 (Bethesda). 2024 Apr 25;14(7):jkae086. doi: 10.1093/g3journal/jkae086 (PMC11228838; doi:10.1093/g3journal/jkae086)
Supplement: jkae086_Supplementary_Data [file jkae086_supplementary_data.zip › Supplementary_File_S2_G3-2024-404838.pdf]

Code Supplement

The genome of the Arctic snow-alga *Limnomonas spitsbergensis* (Chlamydomonadales)

Chris J Hulatt
christopher.j.hulatt@nord.no

Contents

1. Filter PacBio CLR reads to remove organelle sequences
2. Nuclear Genome Assembly
3. Polishing with PacBio reads and Arrow
4. Polishing with Illumina reads and Pilon
5. Polishing with Illumina reads and FreeBayes
6. Genome Curation
7. Repeat Annotation with RepeatModeler2 and RepeatMasker
8. Structural Annotation with BRAKER1, BRAKER2, IsoSeq and TSEBRA
9. Functional annotation
10. Mitogenome preparation

The code provided here are the main commands and options used at various key steps in the genome assembly and annotation, for transparency and reproducibility of the results. Some of the file names and paths have been simplified for clarity, and to run on a cluster environment need to be supplied within an appropriate script.

1. Filtering PacBio CLR reads to remove organelle sequences

Description

The program bam2bam was used to reprocess the sequence reads from each 1M SMRT cell by providing the raw subreads.bam file and scraps.bam from the sequencing run. Optionally specify the library adapters, plus the 2 kb spike-in control sequence and its adapters. Output in various formats is possible-here we use .bam for the next step with blasr, although bam2bam can directly provide .fastq files for assembly as well. The subsequent blasr code example takes all of the PacBio CLR subreads .bam from one sequenced 1M SMRT cell that has previously been subject to control cleaning and adapter removal and aligns them against a pair of draft organelle contigs, collecting all of the unmapped reads from the nuclear genome. Read preparation from raw data with bam2bam and blasr are both available in the command line version of PacBio SMRTLink software. The organelle contigs can be rapidly generated by assembling a 2-5% random subsample of the reads with Canu (see section 10).

Input files

`m54138_190111_194432.subreads.bam` # Pacbio raw subreads.bam from sequence run cell 1
`m54138_190111_194432.scraps.bam` # scraps.bam from the sequence run cell 1
`draft.organelles.fasta` # fasta file containing two draft organelle sequences

Output files

`nuclear.reads.fasta` #fasta file containing all unmapped, nuclear reads

Key software

Version 5.0.1 of SMRTLink was used via command line installation available from PacBio: https://www.pacb.com/support/software-downloads/

Code:

take the raw PacBio sequence reads and remove adapters, internal 2 kb control sequence and its adapters with “bam2bam” in SMRTLink.

```
/path/to/pacbio/smrtcmds/bin/bam2bam --adapter /path/to/adapters.fasta --
controls /path/to/2kb_control.fasta --
controlAdapters=/path/to/control.adapters.fasta -o
/path/to/m54138_190111_194432.clean.control.adapter --nProcs=14
/path/to/m54138_190111_194432.subreads.bam
/path/to/m54138_190111_194432.scraps.bam
```

use further options e.g. --fastq for additional output format.

filter the organelle sequences with “blasr” in SMRTLink.

```
/path/to/pacbio/smrtcmds/bin/blasr
m54138_190111_194432.clean.control.adapter.subreads.bam
/path/to/draft.organelles.fasta --bam --bestn 6 --minMatch 14 --maxMatch 30
--nproc 16 --minAlnLength 3000 --minPctSimilarity 78 --minPctAccuracy 70 --
hitPolicy randombest --out smrt.cell.1.aligned.bam --unaligned
smrt.cell.1.NOT.aligned.fasta
```

Options

`--minAlnLength 3000 --minPctSimilarity 78` #both more conservative than default

The process can be applied to the four sequenced smrt cells:

```
m54138_190111_194432    #cell1
m54138_190112_055912    #cell2
m54138_190223_052854    #cell3
m54138_190223_154036    #cell4
```

concatenate four into one .fasta file

```
cat *.NOT.aligned.fasta > nuclear.reads.fasta
```

2. Nuclear Genome Assembly

Description
This is the Canu assembly command that was used to assemble the nuclear genome on a SLURM cluster distributed across 50 threads using the filtered nuclear reads.

Input files
`nuclear.reads.fasta`

Output files
`Assembly_v8.fasta`

Key software
Canu v.1.7.1 (Koren et al. 2017) referenced in the manuscript and available from: https://canu.readthedocs.io/en/latest/

Code:

```
/path/to/canu-1.7.1/*/bin/canu gnuplotTested=true -d Assembly_v8 -p
Assembly_v8 maxThreads=50 minOverlapLength=2000 minReadLength=2001
corOutCoverage=100 stopOnReadQuality=false genomeSize=277m -pacbio-raw
/path/to/nuclear.reads.fasta
```

Options

`minOverlapLength=2000`	#more stringent than default
`minReadLength=2001`	#more stringent than default
`corOutCoverage=100`	#combined with genomeSize=277m, error correct and trim all reads
`genomeSize=277m`	

Note that using `corOutCoverage=100` with `genomeSize=277m` and about 20 Gb of data, means to check all of the reads. In practice the Canu run log shows about 71-fold achieved coverage. The resulting assembly size was 279.6 Mb.

We can name the assembly `Assembly_v8.fasta`

3. Polishing with PacBio reads and Arrow

Description

The raw Canu assembly .fasta was first polished with the long PacBio CLR reads using the Arrow hidden Markov model. Polishing with long reads removes the majority of small base errors, typically indels, including spanning large repetitive regions. The documented process was repeated three times (3 rounds of Arrow).

Input files

`Assembly_v8.fasta` #raw genome assembly contigs.fasta from Canu
`4cells.merged.cleaned.bam` #a .bam file of the CLR subreads concatenated from 4 smrt cells

Output files

`round3.consensus.fasta` # polished contigs, 3 rounds of Arrow
`round3.consensus.fastq` # polished contigs, 3 rounds of Arrow, with base phred quality scores
`variants.round3.gff` # variants

Key software

Arrow is available via SMRTLink command line installation available from PacBio: https://www.pacb.com/support/software-downloads/. The alignment program "pbmm2" is a PacBio-tailored wrapper around minimap-2 that is available from the Pacific Biosciences git repository: https://github.com/PacificBiosciences/pbmm2

Code:

Index the genome .fasta and align subreads.

```
pbmm2 index Assembly_v8.fasta genome.index.mmi

pbmm2 align genome.index.mmi -j 8 --sort --preset SUBREAD
/path/to/4cells.merged.cleaned.bam aligned.round1.bam
```

index the fasta

```
/path/to/samtools faidx Assembly_v8.fasta
```

sort the bam

```
/path/to/bamtools sort -in aligned.round1.bam -out
aligned.sorted.round1.bam -n 60000
```

n is the number of sequences, per temporary file

make the pbi for the new .bam

```
/path/to/pbindex aligned.sorted.round1.bam

/path/to/arrow aligned.sorted.round1.bam -j8 --coverage 150 -r
Assembly_v8.fasta -o variants.round1.gff -o round1.consensus.fasta -o
round1.consensus.fastq
```

The genome was polished with **THREE rounds of Arrow**. This is simply achieved by recycling the `round1.consensus.fasta` back into the workflow to produce `round2.consensus.fasta` and `round3.consensus.fasta`

4. Polishing with Illumina reads and Pilon

Description
More detailed polishing of non-repetitive regions with Pilon and Illumina PE 250 bp reads.

Input files
`round3.consensus.fasta` #Arrow-polished contigs, 3 rounds
`SR20-23-2_R1.fq` #Illumina paired end 250 bp reads, fastq
`SR20-23-2_R2.fq` #Illumina paired end 250 bp reads, fastq

Output files
`round3.pilon.fasta`

Key Software
The polishing software "pilon" (Walker et al. 2014, cited in the manuscript text) is documented here: https://github.com/broadinstitute/pilon/wiki. The burrows-wheeler read alignment program BWA-MEM (Li 2013, cited in the text) is described here: https://github.com/lh3/bwa

Code:

bwa index the genome

```
bwa index round3.consensus.fasta
```

map reads, convert .sam to .bam and sort

```
bwa mem -t 36 round3.consensus.fasta /path/to/SR20-23-2_R1.fq
/path/to/SR20-23-2_R2.fq > align.bwa.pilon1.sam

samtools view -Sb -@ 36 align.bwa.pilon1.sam > align.bwa.pilon1.bam

samtools sort -@ 30 -o sorted.align.bwa.pilon1.bam align.bwa.pilon1.bam
```

check alignment statistics

```
samtools flagstat -@ sorted.align.bwa.pilon1.bam >
flagstat.align.bwa.pilon1.txt

cat flagstat.align.bwa.pilon1.txt
```

index the bam

```
samtools index sorted.align.bwa.pilon1.bam
```

run Pilon

```
java -Xmx100G -jar /path/to/pilon-1.24.jar --genome round3.consensus.fasta
--frags sorted.align.bwa.pilon1.bam --fix bases --mindepth 20 --outdir
pilon.round1.out --changes --vcf
```

The options `--changes` and `--vcf` record the variants and changes made.

We can name the Pilon-polished genome `round1.pilon.fasta`. As for Arrow, we **re-cycle the process THREE times**, yielding a `round2.pilon.fasta`, and finally a `round3.pilon.fasta`

5. Polishing with Illumina reads and FreeBayes

Description
Final detailed polishing with FreeBayes sensitive variant calling software.

Input files

```
round3.pilon.fasta      #Arrow+Pilon-polished contigs, 3 rounds each
SR20-23-2_R1.fq         #Illumina paired end 250 bp reads, fastq
SR20-23-2_R2.fq         #Illumina paired end 250 bp reads, fastq
```

Output files

```
round5.freebayes.fasta
```

Key software
FreeBayes variant calling software (Garrison and Marth 2012, cited in the manuscript) is available from: https://github.com/freebayes/freebayes. The burrows-wheeler read alignment program BWA-MEM (Li 2013, cited in the text) is described here: https://github.com/lh3/bwa.

Code:

index the fasta

```
bwa index round3.pilon.fasta
```

map reads, convert .sam to .bam and sort

```
bwa mem -t 36 round3.pilon.fasta /path/to/SR20-23-2_R1.fq /path/to/SR20-23-2_R2.fq > align.bwa.freebayes1.sam

samtools view -Sb -@ 36 align.bwa.freebayes1.sam > align.bwa.freebayes1.bam

samtools sort -@ 30 -o sorted.align.bwa.freebayes1.bam align.bwa.freebayes1.bam
```

check alignment statistics

```
samtools flagstat -@ 30 sorted.align.bwa.freebayes1.bam > flagstat.align.bwa.freebayes1.txt
```

Run FreeBayes

```
freebayes -f round3.pilon.fasta -p 1 --standard-filters  --min-coverage 20 sorted.align.bwa.freebayes1.bam > out.freebayes.round1.vcf
```

Run vcftools to filter variants at Q30

```
vcftools --vcf out.freebayes.round1.vcf --out out.freebayes.round1.Q30.vcf
--minQ 30 --recode --recode-INFO-all
```

Reformat the file

```
bgzip -c out.freebayes.round1.Q30.vcf.recode.vcf >
out.freebayes.round1.Q30.vcf.recode.vcf.gz

tabix -p vcf out.freebayes.round1.Q30.vcf.recode.vcf.gz
```

Apply filtered variants to the genome fasta

```
cat round3.pilon.fasta | vcf-consensus
out.freebayes.round1.Q30.vcf.recode.vcf.gz > round1.freebayes.fasta
```

Recycle the workflow **FIVE times** to yield `round2.freebayes.fasta`, `round3.freebayes.fasta`, `round4.freebayes.fasta` and `round5.freebayes.fasta`

The file `round5.freebayes.fasta` is the polished genome assembly.

6. Genome Curation

Description

The polished genome assembly was curated with automated and manual methods. The automated “PurgeHaplotigs” pipeline applied changes by identifying and removing weakly supported contigs that have unusual read coverage. The process substantially reduced the total number of contigs but had a minor effect on the genome size and a negligible effect on BUSCO scores.

Input files

`round5.freebayes.fasta`
`nuclear.reads.fasta`

Output files

`curated.fasta`

Key software

Read remapping was performed with “minimap2” (Li et al. 2018, cited in the manuscript) and available from here: https://github.com/lh3/minimap2. PurgeHaplotigs (Roach et al. 2018, cited in the manuscript) is available from the following link: https://bitbucket.org/mroachawri/purge_haplotigs/src/master/

Code:

Map the PacBio long CLR reads onto the polished assembly and sort the .bam alignment

```
minimap2 -t 8 -ax map-pb round5.freebayes.fasta
~/path/to/nuclear.reads.fasta –secondary=no | samtools sort -m 1G -o
mm2.aligned.bam -T tmp.ali
```

Alignment coverage histogram, choose settings.

```
purge_haplotigs hist -b mm2.aligned.bam -g round5.freebayes.fasta -d 1000 -
t 20
```

Purge haplotigs based on settings from histogram

```
purge_haplotigs  purge  -g round5.freebayes.fasta -c coverage_stats.csv -b
mm2.aligned.bam -t 12
```

We can name the automatically curated genome assembly `genome.curated.fasta` Further quality estimates can be applied, including BUCSO analysis, genome statistics, inspection of removed haplotigs and manual curation examining GC content, size and BLAST results. In practice a few further small contigs were removed from `genome.curated.fasta` by hand, representing the final genome sequence.

7. Repeat Annotation with RepeatModeler2 and RepeatMasker

Description
The following describes the commands used for building a *de novo* repeat library with RepeatModeler2, followed by identifying genomic repeats with RepeatMasker. Note that it can be useful to check and reformat the curated genome .fasta file if needed, for example formatting the contig headers.

Input files
`genome.curated.fasta`

Output files
`curated.repeat-families.fa` #library of consensus sequences for repeat families.
`curated.fasta.masked` #soft-masked genome .fasta

Key software
RepeatModeler2 (Flynn et al. 2020, cited in text) is available as source code or a containerized package under an open license (https://github.com/Dfam-consortium/RepeatModeler, RepeatMasker is detailed at: http://www.repeatmasker.org/).

Code:

```
# make the database

/path/to/RepeatModeler-2.0.2a/BuildDatabase -name repeatmodeler.database
genome.curated.fasta

# run RepeatModeler2 with the LTRharvest pipeline

perl /path/to/RepeatModeler-2.0.2a/RepeatModeler -database genome.curated -
pa 30 -LTRStruct

# run RepeatMasker

RepeatMasker -pa 16 -gff -nolow -xsmall -lib ~/path/to/curated.repeat-
families.fa /path/to/genome.curated.fasta
```

Options

`-xsmall` #soft-mask the genome

RepeatMasker produces a soft-masked copy of the genome that is suitable for downstream annotation with BRAKER, and we can name the final soft-masked genome as `curated.fasta.masked`

Optionally run Tandem Repeat Finder (TRF) and add to the repeat annotations

```
trf genome.curated.fasta 2 7 7 80 10 50 500 -d -m -h
```

Parse the TRF output

```
python3 parseTrfOutput.py --minCopies 1 --gc --statisticsPrefix STATS
genome.curated.fasta.2.7.7.80.10.50.500.dat >
genome.fasta.2.7.7.80.10.50.500.raw.gff
```

note: needed to edit python script csv.field_size_limit(sys.maxsize)

sort and merge the TRF .gff repeat annotations file

```
sort -k1,1 -k4,4n -k5,5n genome.fasta.2.7.7.80.10.50.500.raw.gff > sorted
```

```
bedtools merge -i sorted | awk 'BEGIN{OFS="\t"} {print
$1,"trf","repeat",$2+1,$3,".",".",".","."}' >
genome.fasta.2.7.7.80.10.50.500.merged.gff
```

Apply masking

```
bedtools maskfasta -fi curated.fasta.masked -bed
genome.fasta.2.7.7.80.10.50.500.merged.gff -fo curated.combined.masked -
soft
```

optionally use curated.combined.masked instead of `curated.fasta.masked`

8. Structural Annotation with BRAKER1, BRAKER2, IsoSeq and TSEBRA

Description

The protein-coding genes (CDS) are structurally annotated, producing a .GTF annotation file. Three annotation methods were used including BRAKER1 that takes RNA-Seq evidence from the same organism, BRAKER2 that uses proteins from distant organisms (primarily orthodb) and IsoSeq that are PacBio highly accurate full-length transcripts from the same organism. TSEBRA selects the best gene models, including alternate isoforms, from the three approaches.

Input files

`curated.fasta.masked` #softmasked genome
`S2_02099_1.fq` #RNA-seq reads, paired
`S2_02099_2.fq` #RNA-seq reads, paired
`polished.hq.fastq` #IsoSeq full length transcripts

Output files

`tsebra.gtf` #best set of merged annotations with isoforms

Key software

STAR (Dobin et al. 2013, cited in the manuscript) is documented here: https://github.com/alexdobin/STAR. Braker (Hoff et al. 2016, cited in the manuscript) is documented here: https://github.com/Gaius-Augustus/BRAKER and has multiple further dependencies to install and configure. cDNA Cupcake is documented here: https://github.com/Magdoll/cDNA_Cupcake. minimap2 (Li 2018, cited in the manuscript) is available from here: https://github.com/lh3/minimap2. GeneMarkS-T (Tang et al. 2015, cited in the manuscript) can be obtained from: http://topaz.gatech.edu/GeneMark/license_download.cgi. TSEBRA is the transcript selector for the three annotation methods and the full “long_reads” protocol for iso-seq annotations is available here: https://github.com/Gaius-Augustus/BRAKER/blob/master/docs/long_reads/long_read_protocol.md

Code:

BRAKER1
map the RNA-Seq read evidence to the masked genome using the splice-aware aligner STAR
make STAR index

```
~/path/to/STAR-2.7.3a/bin/Linux_x86_64/STAR --runMode genomeGenerate --
genomeDir ./ --genomeFastaFiles  /path/to/curated.fasta.masked --runThreadN
6 --genomeSAindexNbases 13
```

map reads

```
~/path/to/STAR-2.7.3a/bin/Linux_x86_64/STAR --genomeDir ./ --runThreadN 16
--outFilterMultimapNmax 1 --readFilesIn /path/to/S2_02099_1.fq
/path/to/S2_02099_2.fq --outFileNamePrefix star.masked.out
```

convert sam to bam and sort

```
samtools view --threads 10 -b -o  star.masked.out.bam star.masked.out.sam

samtools sort -m 10G -o sorted.star.masked.out.bam --threads 12
star.masked.out.bam
```

Run braker.pl using the .bam file of RNA-Seq alignments

```
braker.pl --genome= curated.fasta.masked --softmasking --cores=16 --bam=
sorted.star.masked.out.bam --
AUGUSTUS_SCRIPTS_PATH=/path/to/Augustus/scripts --
GENEMARK_PATH=/path/to/GeneMark-ES/gmes_linux_64/ --
workingdir=$wdir/braker1/ 2> $wdir/braker1.log
```

BRAKER2
download viridiplantae ortholog database from orthodb.

```
wget https://v100.orthodb.org/download/odb10_plants_fasta.tar.gz

tar -xzvf odb10_plants_fasta.tar.gz

cat plants/Rawdata/* > odb10.plant.proteins.faa
```

#Download manually from NCBI the Chlamydomonas genome proteins file "GCF_000002595.2_Chlamydomonas_reinhardtii_v5.5_protein.faa"
concatenate all the protein sequences into a single .fasta file

```
cat odb10.plant.proteins.faa
GCF_000002595.2_Chlamydomonas_reinhardtii_v5.5_protein.faa >
all.proteins.faa
```

run BRAKER2 with the "all.proteins.faa" evidence. Paths may vary depending on the installation configuration.

```
braker.pl --genome=curated.fasta.masked --prot_seq=all.proteins.faa --
cores=16 --softmasking --AUGUSTUS_SCRIPTS_PATH=/path/to/Augustus/scripts --
GENEMARK_PATH=/path/to/GeneMark-ES/gmes_linux_64/ --
PROTHINT_PATH=/path/to/ProtHint/bin/ --workingdir=$wdir/braker2/
```

IsoSeq annotaton

```
mkdir $wdir/long_read_protocol

cd $wdir/long_read_protocol
```

Map the IsoSeq transcripts and sort

```
/path/to/minimap2 -t 16 -ax splice:hq /path/to/curated.fasta.masked
/path/to/polished.hq.fastq > long_reads.sam

sort -k 3,3 -k 4,4n long_reads.sam > long_reads.s.sam
```

use cDNA Cupcake to collapse the aligned transcript isoforms

```
collapse_isoforms_by_sam.py --input /path/to/polished.hq.fastq --fq -s
long_reads.s.sam --dun-merge-5-shorter -o cupcake
```

#Run GeneMarkS-T to predict protein-coding regions in the transcripts:

```
/path/to/Augustus/scripts/stringtie2fa.py -g /path/to/ curated.fasta.masked
-f cupcake.collapsed.gff -o cupcake.fa

/path/to/GeneMarkS-T/gmst.pl --strand direct cupcake.fa.mrna --output
gmst.out --format GFF
```

Use the GeneMarkS-T coordinates and the long-read transcripts to create a gene set in GTF format.

```
gmst2globalCoords.py -t cupcake.collapsed.gff -p gmst.out -o
gmst.global.gtf -g /path/to/ curated.fasta.masked
```

Transcript selection with TSEBRA

```
mkdir $wdir/tsebra
cd $wdir/tsebra

/path/to/TSEBRA/bin/tsebra.py -g
/path/to/braker1/augustus.hints.gtf,/path/to/braker2/augustus.hints.gtf -e
/path/to/braker1/hintsfile.gff,/path/to/braker2/hintsfile.gff -l
/path/to/long_read_protocol/gmst.global.gtf -c
/path/to/TSEBRA/config/long_reads.cfg -o tsebra.gtf
```

Again, please note that the file paths and directories will vary depending on your system configuration. The output file from TSEBRA is `tsebra.gtf`. The .GTF file contains the curated set of genes and isoforms based on comparison of BRAKER1, BRAKER2 and IsoSeq annotations above, including all isoforms for a given gene.

9. Functional annotation

Description

Functional annotation of the CDS from TSEBRA. We obtain the protein sequences of the longest isoforms for each gene and then use BLAST, InterProScan and Emapper.py to functionally annotate gene names, domains, function and ontology terms. These can be used individually or collected together. One option to collect them together is to use OmicsBox commercial software.

Input files

```
tsebra.gtf
genome.curated.fasta
```

Output Files

Annotation output from BlastP, InterProScan and Emapper, various formats and integration options are possible.

Key software

BLAST is documented at NCBI: https://blast.ncbi.nlm.nih.gov/doc/blast-help/downloadblastdata.html
InterProScan is documented here: https://interproscan-docs.readthedocs.io/en/latest/
Emapper is documented here: https://github.com/eggnogdb/eggnog-mapper
"agat" and "transeq" are some accessory scripts for handling annotation data, available here: https://github.com/NBISweden/AGAT and from EMBOSS suite via "sudo apt-get install emboss" on the command line. The Augustus/scripts/.. can be found in the Augustus directory that is installed for BRAKER.

Code:

various routes to obtain .gff, longest isoforms, CDS and aa sequences from the tsebra.gtf
convert the tsebra .gtf file to .gff with augustus scripts

```
~/path/to/Augustus/scripts/gtf2gff.pl tsebra.gtf --out=tsebra.gff --gff3
```

use agat to get longest isoform per gene

```
agat_sp_keep_longest_isoform.pl -gff tsebra.gff -o
tsebra_longest_isoform.gff
```

augustus script again to get the .fasta coding sequences

```
~/path/to/Augustus/scripts/getAnnoFasta.pl tsebra_longest_isoform.gff –
seqfile=curated.fasta.masked
```

protein sequences can be extracted

```
~/path/to/Augustus/scripts/Augustus/scripts/gtf2aa.pl curated.fasta.masked
tsebra.gtf prot.fa
```

translate only the longest transcripts

```
transeq -sequence tsebra_longest_isoform.codingseq -outseq
tsebra_longest_isoform.AAtrans.fasta
```

Functional annotation with BlastP, InterProScan-5 and Emapper.py
remove the "*" stop in the aa sequences

```
sed '/^>/! {s/\*//g}' tsebra_longest_isoform.AAtrans.fasta >
proteins.no.dot.fasta
```

BLAST

```
mkdir BlastP
cd BlastP

blastp -query ../proteins.no.dot.fasta -db /home/chris/Documents/blast-
SwissProt-ncbi/swissprot -outfmt 5 -evalue 1e-5 -word_size 3 -show_gis -
num_alignments 20 -max_hsps 20 -out blastp.swissprot.braker2.annot.out_e-
5.xml -num_threads 20
```

Note here we use an e-value threshold of 1e-5, and the SwissProt database.
The file output format "-outfmt 5" is for importing into OmicsBox or Blast2GO.

#InterProScan

```
cd ..
mkdir interproscan
cd interproscan

~/path/to/interproscan-5.53-87.0/interproscan.sh --goterms --pa -iprlookup
-i ../proteins.no.dot.fasta -b interproscan.proteins -cpu 20
```

Emapper

```
cd ..
mkdir emapper
cd emapper

~/path/to/emapper.py --database euk -i ../proteins.no.dot.fasta --output
eggNOG-mapper.euk -m diamond
```

using DIAMOND blast option for speed

The results of these three functional annotation methods (BlastP, InterProScan and Emapper.py) can be imported into OmicsBox or Blast2GO, which provides convenient methods for merging, validating and refining non-redundant annotations and gene ontology terms. Other software-independent formats are equally possible.

10. Mitogenome preparation

Description

The organelles were efficiently assembled to single contigs using small random subsets of PacBio reads. The following commands were used for assembly and polishing of the complete circular mapping mitogenome presented in the manuscript.

Input files

`subsampled.reads.5.percent.fasta` #fasta containing 5% of the total PacBio long reads

Output files

`trimmed.asm.v2.5percent.arrow.r2.polished.500x.fasta`

Key software

Canu v.2.0 (Koren et al. 2017) is referenced in the manuscript and available from: https://canu.readthedocs.io/en/latest/. The alignment program "pbmm2" is a PacBio-tailored wrapper around minimap-2, and is available from Pacific Biosciences git repository: https://github.com/PacificBiosciences/pbmm2 The GenomicConsesnsus has been largely superseded: https://github.com/PacificBiosciences/gcpp but is available in older versions of SMRTLink: https://www.pacb.com/support/software-downloads/

Code:

Assemble a small 5% subset of total reads with Canu 2.0

```
/path/to/canu-2.0/Linux-amd64/bin/canu -d ~/path/to/Canu.asm/ -p subsample5
genomeSize=20m minReadLength=10000 saveReads=true minOverlapLength=2000
minInputCoverage=5 stopOnLowCoverage=5 -pacbio-raw
/path/to/subsampled.reads.5.percent.fasta
```

Mitogenome contig is identified and trimmed slightly at the ends, many kb overlap remains.
name the trimmed contig "trimmed.asm.v2.5percent.fasta".
apply "genomic consensus" for base polishing by aligning with pbmm2 and polishing with arrow.

```
pbmm2 index trimmed.asm.v2.5percent.fasta trimmed.asm.v2.5percent.mmi

pbmm2 align --sort -j 16 --preset SUBREAD -l 3000
trimmed.asm.v2.5percent.mmi
/path/to/m54138_190223_154036.clean.control.adapter.subreads.bam
trimmed.asm.v2.5percent.aligned.bam
```

```
pbindex trimmed.asm.v2.5percent.aligned.bam

samtools faidx trimmed.asm.v2.5percent.fasta

variantCaller --algorithm arrow -j 16 trimmed.asm.v2.5percent.aligned.bam -
r trimmed.asm.v2.5percent.fasta -o
trimmed.asm.v2.5percent.arrow.r1.polished.fasta -o
trimmed.asm.v2.5percent.arrow.r1.polished.fastq -o
trimmed.asm.v2.5percent.arrow.r1.polished.varaints.vcf
```

re-polish a second time with arrow and more depth (500 x consensus depth this time)

```
pbmm2 index trimmed.asm.v2.5percent.arrow.r1.polished.fasta
trimmed.asm.v5.5percent.arrow.r1.polished.mmi

pbmm2 align --sort -j 16 --preset SUBREAD -l 4000
trimmed.asm.v5.5percent.arrow.r1.polished.mmi
/path/to/m54138_190223_154036.clean.control.adapter.subreads.bam
trimmed.asm.v2.5percent.arrow.r2.polished.aligned.bam

pbindex trimmed.asm.v2.5percent.arrow.r2.polished.aligned.bam

samtools faidx trimmed.asm.v2.5percent.arrow.r1.polished.fasta

variantCaller --algorithm arrow -j 16 --coverage 500
trimmed.asm.v2.5percent.arrow.r2.polished.aligned.bam -r
trimmed.asm.v2.5percent.arrow.r1.polished.fasta -o
trimmed.asm.v2.5percent.arrow.r2.polished.500x.fasta -o
trimmed.asm.v2.5percent.arrow.r2.polished.500x.fastq -o
trimmed.asm.v2.5percent.arrow.r2.polished.varaints.500x.vcf
```

The final mitogenome polished a second time at 500 x depth is found in `trimmed.asm.v2.5percent.arrow.r2.polished.500x.fasta` that can be manually circularized, checked and annotated.
